# Perceived dyspnea and experience of hospitalized patients with acute decompensated heart failure undergoing an early MObilization protocol with immersive Virtual rEality: MOVE study protocol for a parallel superiority randomized clinical trial

**DOI:** 10.1186/s13063-023-07786-z

**Published:** 2023-11-24

**Authors:** Iasmin Borges Fraga, Larissa Gussatschenko Caballero, Pedro Dal Lago, João Lucas Campos de Oliveira, Marina Scherer, Mauren Porto Haeffner, Eneida Rejane Rabelo-Silva

**Affiliations:** 1 Graduate Program in Cardiology and Cardiovascular Sciences, Universidade Federal Do Rio Grande Do Sul, Porto Alegre, Brazil; 2 Graduate Program Program of the School of Nursing, Universidade Federal Do Rio Grande Do Sul, Porto Alegre, Brazil; 3 Cardiology Division and Heart Failure Clinic, Hospital de Clínicas de Porto Alegre, Porto Alegre, Brazil; 4 Graduate Program in Rehabilitation Sciences, Universidade Federal de Ciências da Saúde de Porto Alegre, Porto Alegre, Brazil

**Keywords:** Randomized clinical trial, Heart failure, Early mobilization, Cardiac rehabilitation, Virtual reality, Exercise tolerance, Patient experience

## Abstract

**Background:**

Immersive virtual reality (VR) is an innovative strategy for inpatient rehabilitation programs. Using immersive VR in early mobilization protocols has not yet been investigated in the setting of hospitalized patients with acute decompensated heart failure (ADHF), especially to improve perceived dyspnea, a common symptom of heart failure (HF).

**Methods:**

This is a single-center parallel superiority randomized clinical trial. The study will be conducted at a public teaching hospital in Brazil from January 2023 to January 2024. The sample will include adult patients with ADHF hospitalized for at least 24 h, randomly assigned in a 1:1 ratio to the control (standard early mobilization protocol conducted in the intensive care unit (ICU)) or intervention group (the same standard early mobilization protocol but associated with immersive VR). The primary outcome will be assessing perceived dyspnea, and the secondary outcome will be assessing patient experience.

**Discussion:**

Using immersive VR in early mobilization protocols in the ICU is expected to improve perceived dyspnea in patients with ADHF as well as patient experience regarding care. This study has the potential to increase patient adherence to early mobilization protocols in the setting of ADHF as well as to promote a positive patient experience. Filling this gap could promote the rational incorporation of technologies in health care.

**Trial registration:**

This study protocol is in its first version. ClinicalTrials.gov NCT05596292. Registered on 1 December 2022.

**Supplementary Information:**

The online version contains supplementary material available at 10.1186/s13063-023-07786-z.

## Introduction

HF is a highly prevalent clinical syndrome in which patients experience frequent episodes of decompensation that lead to periods of clinical instability and the need for admission to an ICU [1]. Repeated hospitalizations contribute to functional and cardiac decline [2, 3], and these deleterious effects are closely linked with previous conditions, especially in patients with a chronic disease that is difficult to manage [4].

Treatment in a critical setting often involves prolonged bed rest, imposing a series of limitations on daily activities. The prolonged use of pharmacological agents, diet changes, worsening of the disease, and physical inactivity further impair patients’ health, favoring the worsening of quality of life (QoL) [5]. Conventional methods of early rehabilitation, such as progressive walking, calisthenics and cycle ergometer use, have demonstrated their capacity to mitigate these negative consequences [6]. However, their application might be limited by patient discomfort, fatigue, dyspnea, or other clinical conditions. Thus, in recent years, alternatively to traditional inpatient rehabilitation programs, more attractive methods have been investigated aiming to improve patient experience and increase patient engagement during exercise. Immersive VR has a prominent role among these new technologies [7]. It blocks unpleasant stimuli and distracts patients from exercise-related stress, helping them manage common negative symptoms such as fatigue and dyspnea during physical exercise [8] and providing a break from the hospital environment [9].

Dyspnea is one of the main limiting symptoms in patients with HF. It is essential that both the subjective perception of breathlessness and the underlying physiological disturbances be considered [10]. Interventions should be designed with an understanding of this complex interplay, aiming to provide relief by targeting both aspects. In patients with ADHF, dyspnea is not just a symptom but a significant impediment to their rehabilitation and overall well-being. Traditional interventions, while beneficial [6], may not always offer immediate or substantial relief from the intense discomfort of dyspnea. This is where immersive virtual environments offered by VR come into play. VR can potentially redirect the patient’s focus from their discomfort, immersing them in a distraction that traditional methods might not provide [9]. By doing so, VR can alleviate the immediate sensation of breathlessness and provide a therapeutic avenue that complements traditional approaches.

In critical care settings, where dyspnea can delay or even interrupt inpatient rehabilitation, the rapid relief offered by VR might make it a superior option. Thus, while both traditional and VR interventions have their merits, the immersive and immediate nature of VR gives it a distinctive edge in addressing the challenges posed by dyspnea in patients with ADHF. However, despite VR studies with other populations [7, 9], studies in patients with HF are still scarce.

To date, no studies with a robust evidence level have investigated the safety of early mobilization in patients with ADHF admitted to ICU-level care. Therefore, further research should assess the feasibility and safety of early interventions, which may promote a more effective recovery and reduce hospitalization-related risks, as is already better established in the literature in other populations [11, 12]. Also, the distraction of VR can lead to reduced perception of dyspnea, which, in turn, can help provide a better experience during hospitalization. To the best of our knowledge, no published study assesses the improvement of parameters such as perceived dyspnea in patients with ADHF undergoing an immersive VR-assisted early mobilization protocol, nor aims to understand patient experience with this technology.

Filling this gap could improve the rational and direct incorporation of new technologies in health care, allowing for better outcomes in this clinically vulnerable population. This randomized clinical trial hypothesis is that patients hospitalized due to ADHF undergoing an early mobilization protocol with immersive VR will have improved perceived dyspnea as well as a positive patient experience with the technology compared with those undergoing a conventional mobilization protocol without immersive VR.

## Methods

### Study setting

**Fig. 1:**
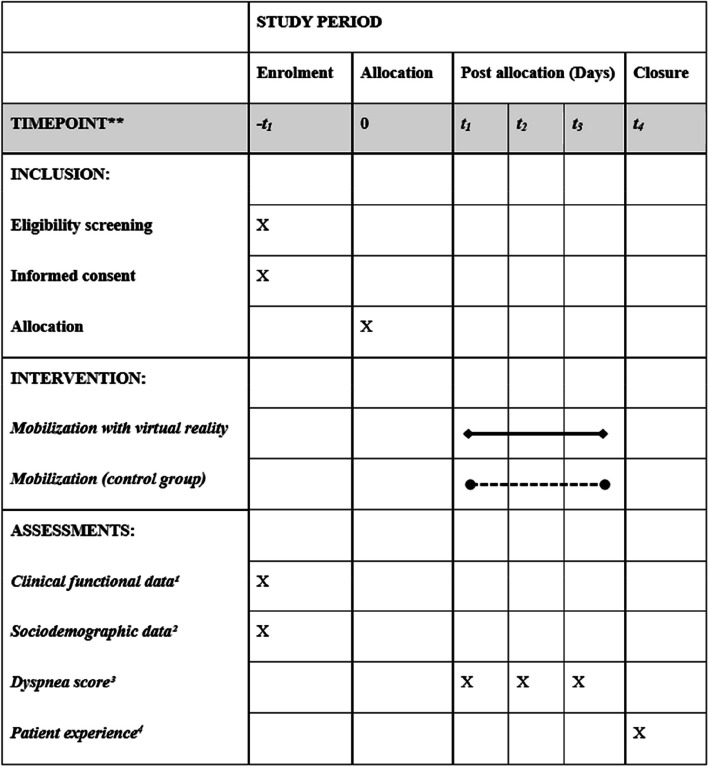
Timetable for inclusions, interventions, and assessments. ^1^Risk of falls accessed by Severo-Almeida-Kuchenbecker 2 score and functional independence accessed by Barthel scale. ^2^Age, sex, education, current and previous hospitalization, disease condition, associated comorbidities, and medication use. ^3^Borg Rating of Perceived Exertion Scale. ^4^Net Promoter Score, Likert scale, and interview

This is a single-center parallel superiority randomized clinical trial blinded for primary outcome collection and data analysis. The study will be conducted at the Hospital de Clínicas de Porto Alegre (HCPA), a public teaching hospital in Brazil, from January 2023 to January 2024. This study protocol is in its first version and was registered at ClinicalTrials.gov under identification number NCT05596292 and is in accordance with the Standard Protocol Items: Recommendations for Interventional Trials (SPIRIT) [13] (Fig. 1). The SPIRIT Checklist for Trials is in the Additional file 2.

The Research Ethics Committee of the HCPA and the Universidade Federal do Rio Grande do Sul (UFRGS) approved this study before data collection, under registration number 62209822.7.0000.5327. The research team will be responsible for the integrity of this study.

### Participants

Patients admitted to the hospital’s ICU with a medical diagnosis of ADHF will be included in the study. The medical diagnosis will be made by the hospital team at ICU admission, based on anamnesis, symptomatology, and necessary complementary tests (ejection fraction verified by echocardiography). The research team is not involved in the diagnosis.

**Fig. 2:**
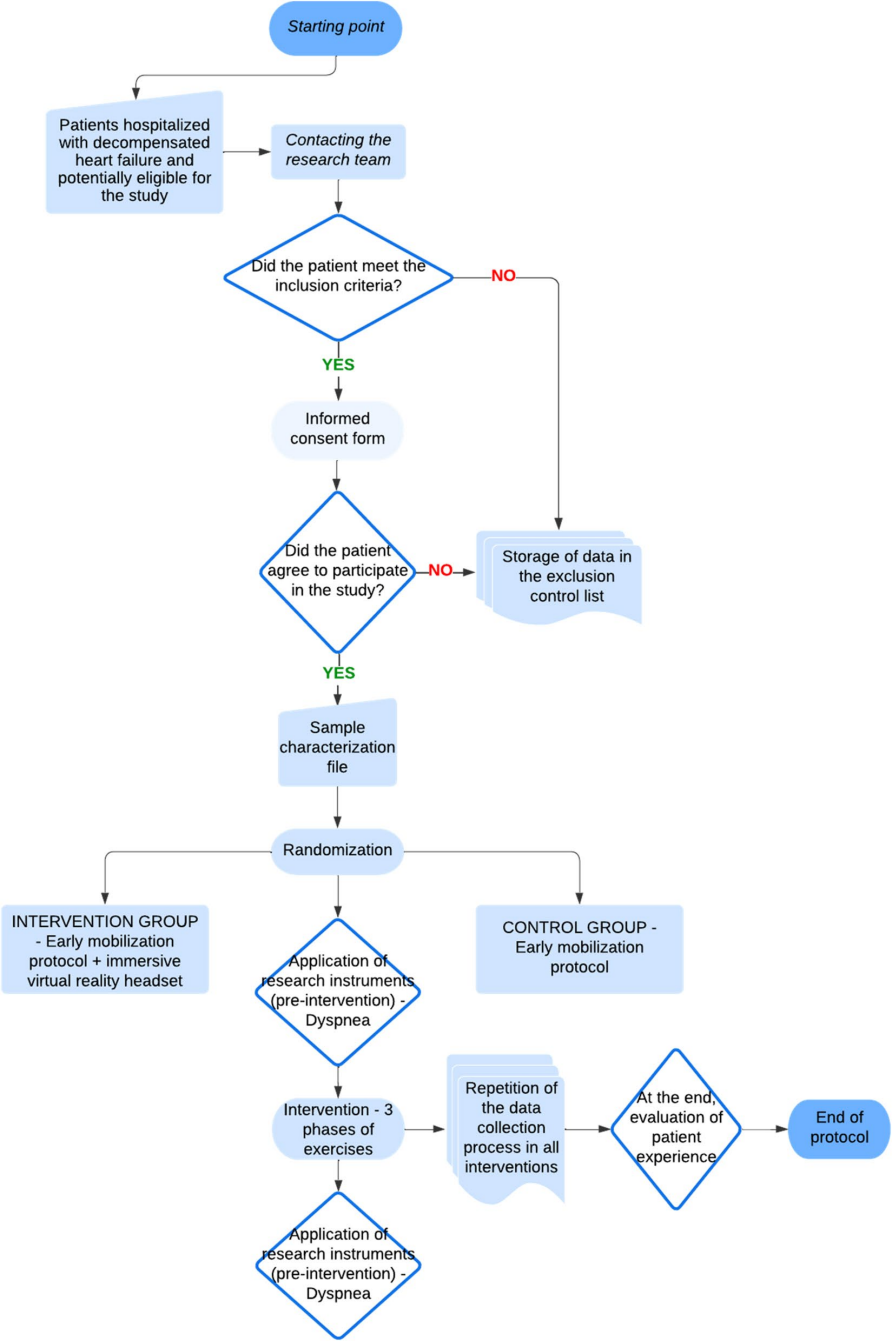
Description of the study protocol. Source: Research team

Participants will be randomly assigned to two groups, as shown in Fig. 2:

1. Control group (CG): participants will undergo three sessions of conventional treatment consisting of an inpatient early mobilization protocol conducted in the ICU.
2. Intervention group (IG): participants will undergo three sessions of conventional treatment consisting of an inpatient early mobilization protocol conducted in the ICU with the use of an immersive VR headset.

### Eligibility criteria

#### Inclusion criteria

- 18 years or older.
- Diagnosis of ADHF in the medical records.
- Be lucid and coherent.
- Be hospitalized for at least 24 h.
- Have the authorization of the medical team for inclusion.
- Patients with intermittent noninvasive ventilation can be included in the study and undergo the early mobilization protocol in the intervals of this therapy.

#### Exclusion criteria

- Patients with invasive mechanical ventilation or circulatory support (extracorporeal membrane oxygenation).
- Patients with neurodegenerative diseases.
- Pregnant patients.
- Patients with hemodynamic instability (characterized by symptomatic hypotension with systolic blood pressure equal to or less than 80 mmHg and diastolic blood pressure equal to or less than 50 mmHg, heart rate above 130 bpm or below 40 bpm at rest, oxygen saturation below 90% with spontaneous ventilation or oxygen support).
- Patients with modified Borg scale score ≥ 4 at rest.
- Patients with difficulty communicating.
- Patients who cannot adapt to wearing an immersive VR headset.

### Intervention and groups

*CG:* participants in the CG will undergo a maximum of three standard early mobilization sessions in the ICU, with different exercise phases, as shown in Table 1.

**Table 1:** Early mobilization protocol phases

Stage	Intervention
Day 1	In-bed cycle ergometry in sitting position, 5 min for upper limbs and 5 min for lower limbs
Day 2	Day 1 + training in orthostatic position
Day 3	Walking for 5 to 10 min

Source: Early mobilization protocol adapted from Delgado et al. [6]

*IG:* participants in the IG will undergo a maximum of three standard early mobilization sessions in association with the use of an immersive VR headset (VR Shinecon G10), with different exercise phases, as previously described. Immersive VR headsets are compatible with smartphones (Samsung A30), allowing patients to interact with a 360-degree YouTube video (https://youtu.be/h_0oimKWlNs) while listening to lo-fi music on Spotify (https://open.spotify.com/track/7qcWkecFox8uO6mYyYwpy6?si=qDYj9_kpSCiPsXNRirFrYQ) through wireless earbuds (Multilaser Joy PH308 Bluetooth sports headphone with microphone).

Tolerance will be considered and assessed using the Borg scale, vital signs, and the patient’s response to the following question: “In your opinion, were you able to tolerate this exercise well?” (yes or no).

Participants who can tolerate the exercise at a given stage may move on to the next stage in the same session. The participant will repeat the same exercise from the previous stage before performing the next exercise. If the patient fails to complete one of the phases, the intervention can be interrupted to respect the patient’s tolerance. Likewise, if the patient initiates but fails to complete the protocol on the second day, a new attempt will be made on the third day, beginning on the stage the patient was previously on.

In addressing the rationale for the intervention dosage in both groups, it is paramount to understand the patients’ profiles. Patients with ADHF often experience significant dyspnea at rest and demonstrate low exercise tolerance. By the time the early mobilization protocol is initiated, after 24 h of hospitalization and with the approval of the medical team, patients have usually achieved a level of stability. However, they are still likely to have symptoms that restrict their participation in conventional exercise programs.

All participants will have individual rooms, and the ambulation stage of both groups will be performed in a parallel corridor, where seeing the other patients hospitalized in the unit is impossible, ensuring their privacy.

### Outcomes

The primary outcome is the assessment of perceived dyspnea in all sessions in both groups using the modified Borg scale [14].

The secondary outcome is the assessment of patient experience at the end of the early mobilization protocol with and without immersive VR using the Net Promoter Score (NPS) tool and the Likert scale [15] as well as an open-ended interview.

Other variables include (a) assessment of vital signs, (b) collection of sociodemographic data, (c) assessment of risk of falls, and (d) assessment of functional independence. To assess the risk of falls, the standard Severo-Almeida-Kuchenbecker (SAK) 2 scale [16] will be used. To assess functional independence prior to hospitalization, the Barthel scale [17] will be used. Both scales are validated.

### Participant timeline and study protocol

The study protocol is shown in Fig. 2. The informed consent will be obtained by one of the researchers. The study begins with the daily screening of hospitalized patients with HF, performed 7 days per week, in the morning and afternoon shifts, using the electronic access to patients admitted to the ICU. Baseline measurements will be performed before randomization, when the patient has potential eligibility to be included, is cleared to start early mobilization, and has accepted.

Patients with potential eligibility, but who for any clinical reason were unable or refused to participate in the study, will be included in a screening exclusion worksheet with coded patient identification data, the date, and the reason for non-inclusion to later report this information.

The team responsible for conducting the intervention and collecting variables will receive training from the research team prior to collection, and patients will be monitored daily by the research team while in the study protocol. The study period will be 1 to 3 days, during this phase, patients will be subjected exclusively to the mobilization protocol proposed by the study. Therefore, the patient should not do early mobilization exercises with the hospital physical therapy team.

However, patients in both groups are allowed to do breathing exercises, get out of bed, and walk to perform daily activities (such as bathing, sitting in a chair, etc.) with the multidisciplinary team (nursing or physical therapy), since the sessions with the research team last about 40 min and the patient would not benefit from staying in bed for 24 h. After the study period, the health care team will continue to treat patients according to standard practice or hospital protocol.

After randomization, the first patient contact will include an assessment of perceived dyspnea according to the modified Borg scale [14] and vital signs at rest and immediately after the assessment. In the first day, patients will perform 10 min of in-bed cycle ergometry (5 min for upper limbs and 5 min for lower limbs). After a 24- to 30-h break, perceived dyspnea and vital signs will be reassessed, and the patient will perform training in orthostatic position in addition to in-bed cycle ergometry. On the last day of intervention, 24 to 30 h after the previous intervention, variables will be reassessed again pre- and post-intervention and the patient will walk for 5 to 10 min.

Cardiovascular rehabilitation in the hospital phase aims to optimize both physical and psychological outcomes for the patient. The goal at this juncture is not necessarily intensive rehabilitation, but to initiate a process that ensures patient safety and comfort while also laying the groundwork for post-discharge activities. At this stage, mild-intensity exercises are deemed optimal [18], and the proposed timings sufficiently meet this intensity. Monitoring of vital signs and of the patient’s perception of exertion is essential to maintaining this balance.

All interventions will be performed with or without the use of immersive VR headsets, videos, and earbuds, as previously described. To assess patient opinion at the end of interventions, the NPS tool and the Likert scale [15] will be used. In addition, an open-ended interview will be conducted to further explore quantitative results related to patient experience regarding the proposed interventions in both groups.

Regarding adherence to the study protocol, the research team highlights that mobilization therapy is recommended in the usual treatment of hospitalized patients with ADHF. The proposed exercise protocol does not differ from the mobilization therapy currently offered by the hospital care team; the only difference is the impact evaluation of using VR during mobilization. If patients accept to participate, the research team will explain to both groups that the study requires some additional tests before starting the mobilization protocol, immediately after, and up to 2 days at the end of the protocol. The research team is responsible for reinforcing the importance of the mobilization protocol and the test to participate in the study.

### Sample size

The study by Nomura et al. [19] used the Borg scale in the first 24 h to assess the effects of medication on the sensation of dyspnea and other outcomes. In the first 24 h, the patient’s sensation of dyspnea significantly improved, with a significant reduction in the Borg scale score [19].

Initially, applying the Borg scale obtained an index of 7. After intervention, the index ranged from 2 to 3. This study showed that after the first 24 h the patient tends to have less changes in the dyspnea sensation, given the use of medication [19]. Based on the literature, we believe that 1 point is a considerable change score, since the patient would probably not have a more significant change due to the compensation of the clinical condition.

The sample size was estimated to detect differences in mean dyspnea between the intervention and control groups using the Borg scale, with a difference of 1 point being relevant for the study. For this purpose, the online version of the power and sample size (PSS) Health tool was used [20]. Considering an 80% power, a 5% significance level, and a 1.6-point standard deviation [19, 21], a total sample size of 54 participants was calculated, with 27 participants in each group. To account for 20% of possible losses and refusals, the total sample size will need to include 66 participants (33 in each group). Seeking to reach the appropriate sample number to conclude the study, a daily screening will be carried out by the research team of patients meeting the inclusion criteria admitted to the study site.

### Recruitment

Potential eligible participants will be recruited by an active daily electronic search of patients admitted to the ICU. The research team will be available in the morning and afternoon shifts. All potentially eligible participants will be screened and, if they meet the inclusion criteria, will be approached in the inpatient unit.

### Randomization and allocation sequence

The Research Electronic Data Capture® (REDCap®) software will be used for randomization. After agreeing to participate in the study, patients will sign an informed consent form. The research team will add in REDCap® the following information: sociodemographic data (age, schooling level, profession, religion, etc.) and clinical data (disease profile, New York Heart Association [NYHA] classification, previous hospitalizations in the last 12 months, other diseases, HF etiology, application of the Borg, SAK 2, and Barthel scales). After all variables are included, the electronic platform randomizes the patient. The allocation sequence is a randomization based on a single 1:1 sequence.

### Blinding

Patient randomization will be conducted by a researcher not involved in data collection. Physical therapists will be blinded for the collection of the outcomes (perceived dyspnea and patient experience) and data analysis. Data will be collected by researchers uninvolved in the intervention but who are not blinded to group allocation due to the nature of the protocol and the outcomes. Data analysis will be performed by a statistician independent from the study.

### Study variables

- Dyspnea: dyspnea will be assessed by the modified illustrated Borg scale [14]. One of the researchers responsible for collecting data will ask patients to assess the degree of perceived dyspnea at rest and immediately after the intervention in all interventions according to a list of figures that reflect a numerical order from 0 to 10. In it, 0 corresponds to no dyspnea, 0.5–2 corresponds to minor dyspnea, 3–4 corresponds to mild dyspnea, and 5–10 corresponds to severe dyspnea [22].
- Patient experience: patient experience will be assessed by the NPS tool and the Likert scale. The NPS is intended to measure the satisfaction level of customers or patients regarding a product or service. The scores are assigned on a numerical scale from 0 to 10 and are divided into three categories: detractors (0 to 6), passives (7 or 8), and promoters (9 or 10) [15]. The Likert scale consists of psychometric responses commonly used in opinion surveys when responding to a questionnaire. Respondents specify their level of agreement with a statement or question as follows: strongly disagree, disagree, neutral, agree, and strongly agree. An open-ended interview will also be conducted at the end of the intervention by a researcher who is not involved in the application of the early mobilization protocol to preserve the subjectivity associated with patient experience.
- Risk of falls: participants must meet the eligibility criteria before being enrolled in the study. The risk of falls will be assessed with the SAK 2 scale. The scale assesses the following variables: disorientation/confusion, frequent urination, difficulty walking, lack of caregiver, postoperative period, number of medications administered before the fall (up to 72 h), and previous falls. The score ranges from 0.5 to 17 points, divided into low (≤ 6.0), moderate (6.5–10), or high (≥ 10.5) risk of falls. This scale is systematically used at HCPA and is part of the daily assessment conducted by the nursing team; the results are recorded in the patients’ medical records [16].
- Sociodemographic data: if the participant meets the eligibility criteria and agrees to participate in the study, sociodemographic data will be collected before randomization using a questionnaire including questions about age, gender, education, occupation, current and previous hospitalizations, disease and associated comorbidities, and use of medications.
- Functional independence: functional independence will be assessed before randomization by the Barthel scale, which considers the degree of help needed for feeding, bathing, activities of daily living, dressing, bowel control, bladder control, toilet use, transfers, mobility, and stairs. The score ranges from 0 to 100, and the higher the score, the greater the functional independence and the less the need for help to perform activities of daily living [17].

### Data collection

The primary outcome should be assessed immediately before and after each mobilization therapy session, less than 5 min before the start and immediately after the completion of the mobilization protocol.

At the end of the interventions, patient experience will be assessed in both groups. Patients included in the study will be contacted in the ICU within 48 h of the end of the intervention and will be invited to answer questions related to their experience during the mobilization protocol with and without the use of immersive VR.

The outcomes will be collected by two researchers previously trained in the pilot study. The researchers involved in data collection are not involved in applying the study protocol and are not blinded to group allocation due to the nature of the protocol and the outcomes assessed, such as patient experience.

### Data management

In both groups, patients will be treated according to the hospital’s protocol of good safety practices. Any solicited and spontaneously reported adverse events (such as loss of venous access or probes, falls, nausea or dizziness, or symptomatology of hemodynamic instability) will be recorded for further reporting.

Data will be stored in the REDCap® platform. Only researchers trained on this software and in the pilot study will collect data. Data will only be extracted from the REDCap® platform after the inclusion and establishment of the total number of participants. Data of excluded patients will be stored in an exclusion control list for future data reporting.

### Statistical analysis

The database will be built on the REDCap® platform. All data will be analyzed on Statistical Package for the Social Science (SPSS) version 22.0 for Windows®. The Shapiro–Wilk test will be used to assess data normality. For quantitative data, categorical variables will be expressed as absolute and relative frequencies, and continuous variables will be expressed as means and standard deviations or medians and interquartile ranges.

To address the statistical analysis of multiple time point measurements for the primary outcome intragroup and intergroup, repeated measures tests will be done using Generalized Estimating Equations (GEE). Also, associations between vital signs and perception of dyspnea will be analyzed by the GEE test too.

Chi-square will be used for association of categorical variables, and analysis of variance (ANOVA) or Kruskal–Wallis to compare the categorized sensation of dyspnea with the other variables. Depending on variable behavior or characteristic, the correlations will be analyzed by the Pearson or Spearman coefficient. The significance level will be set at 5% (*p* < 0.05). All analyses will be performed by intention to treat.

Information obtained from the Likert scale and the NPS tool will be analyzed using resources from the NVivo software version 12 for Windows®, which combines the main tools required for working with texts, mixed methods, and data from the literature.

### Potential biases

Participants will not be blinded to their allocation group. The researchers responsible for the intervention in both groups will not be involved in collecting primary and secondary outcomes. The researchers responsible will not be blinded to collecting outcomes due to the relationship between the use of immersive VR, clinical results, and patient experience.

## Discussion

The clinical condition of patients with HF worsens with each hospitalization [23]. Considering the deleterious effects of bed restriction, inpatient rehabilitation programs that seek to reduce periods of immobility are important for this population [18].

The physiological rationale behind early mobilization, whether achieved by conventional or VR-enhanced methods, and its impact on dyspnea in individuals experiencing ADHF are a matter of significant importance. Understanding how such interventions can positively influence experience and physical conditions, especially during the critical phase of ICU admission, warrants thorough investigation.

In this sense, new technologies available on the market may be implemented to help patients adhere to and tolerate early mobilization protocols during ICU stay. The advantage of using immersive VR in inpatient rehabilitation processes is that it can provide a break from the hospital environment, allowing patients to feel more comfortable when performing exercises and reducing the frequency of undesirable symptoms [8, 9]. In situations where conventional physical rehabilitation is hindered by dyspnea-related limitations, VR could provide an innovative solution, and it may also improve patients’ experience of the care process.

Rigorous research is warranted to obtain a comprehensive understanding of the impact of VR on dyspnea in ADHF scenarios. By engaging patients in movement and activity at an early stage, it may be possible to counteract the deleterious effects of immobility that often accompany hospitalization [18].

Thus, this study is relevant due to its potential to increase patient adherence to mobilization protocols during hospitalization and, consequently, contribute to a positive personal experience. Furthermore, given the frequent readmissions and how the limitations imposed by the disease can reduce QoL, it is important to have strategies that seek a more motivating and humanized treatment. Filling this gap could promote the rational and direct incorporation of technologies in health care, allowing possible favorable outcomes for this clinically vulnerable population.

Using immersive VR in interventions may pose some risks to patients, since patients admitted to an ICU are fragile and susceptible to falls and therefore require supervision. Also, to introduce this technology in treatment strategies, patients must first adapt to using VR headsets, which is a possible barrier for conducting this study.

## Trial status

This study protocol is in its first version, registered in ClinicalTrials.gov on 01/12/2022. The recruitment started on January 2023 and the approximate date of its completion is in January 2024. Any necessary change in this protocol will be informed to the Research Ethics Committee of UFRGS and HCPA. All authors will have access to the final trial dataset.

### Supplementary Information

**Additional file 1.** Informed consent form.

**Additional file 2.**

## Data Availability

The results of this research will be published in a peer-reviewed journal and the participant-level data and code will be public.

## Abbreviations

ADHF: Acute decompensated heart failure
CAPES: Coordination for the Improvement of Higher Education Personnel
CNPq: National Council for Scientific and Technological Development
HF: Heart failure
VR: Virtual reality
ICU: Intensive care unit
QoL: Quality of life
HCPA: Hospital de Clínicas de Porto Alegre
SPIRIT: Standard Protocol Items: Recommendations for Interventional Trials
UFRGS: Universidade Federal do Rio Grande do Sul
NPS: Net Promoter Score
SAK: Severo-Almeida-Kuchenbecker 2 scale
CG: Control group
IG: Intervention group
PSS: Power and sample size
REDCap®: Research Electronic Data Capture®
NYHA: New York Heart Association
SPSS: Statistical Package for the Social Science
GEE: Generalized Estimating Equations
ANOVA: Analysis of variance
FIPE: Research Incentive Fund of the Hospital de Clínicas de Porto Alegre
