# Supplementary material for: Perceived dyspnea and experience of hospitalized patients with acute decompensated heart failure undergoing an early MObilization protocol with immersive Virtual rEality: MOVE study protocol for a parallel superiority randomized clinical trial
Source: Trials. 2023 Nov 24;24:751. doi: 10.1186/s13063-023-07786-z (PMC10675897; doi:10.1186/s13063-023-07786-z)
Supplement: Supplementary file 1 — Additional file 1. Informed consent form. [file 13063_2023_7786_MOESM1_ESM.docx]

**Appendices**

**Model of consent form**

**INFORMED CONSENT FORM**

**MOVE PROJECT**

**CAAE Project No. 62209822700005327**

**Project Title:** Perceived dyspnea and experience of hospitalized patients with acute decompensated heart failure undergoing an early **MO**bilization protocol with immersive **V**irtual r**E**ality: **MOVE**study protocol for a parallel superiority randomized clinical trial

You are invited to participate in a study that aims to evaluate the effect of using virtual reality glasses on the sensation of dyspnea/shortness of breath during exercise practice, and what this new experience was like. This study is being conducted by the Coronary Care Unit (UCC) of the Hospital de Clínicas de Porto Alegre (HCPA).

If you accept this invitation, your involvement will include participating in three sessions of an exercise program, performing activities including riding a mini stationary bike and walking, with or without having virtual reality glasses on, depending on the group you are allocated into. The intensity of the exercises will progress according to your tolerance. We stress that exercising during hospitalization is a routine in the unit where you are hospitalized, and you will be followed and guided by qualified professionals at all times. If you accept this invitation, we will assess the sensations of shortness of breath and fatigue and your ability to perform everyday tasks. Moreover, you will answer questions about your opinion on the proposed topic, on a personal basis. We also need your authorization to access your medical record and consult personal information and other clinical data that may be relevant.

The possible risks resulting from your participation in this study include difficulty in adapting to the virtual reality glasses, and you may also feel uncomfortable due to the length of the questionnaire or the content of its questions, which involve some aspects of your intimacy.

The possible benefits from your participation are undertaking a new experience and guided exercise practice. Your participation will also contribute to improve the knowledge on the topic studied and, if applicable, may benefit future patients.

Your participation in the study is completely voluntary, that is, it is not compulsory. If you decide not to participate or withdraw your consent, the care you currently receive or may receive in the future at the institution will not be affected.

Your participation in the study will not result in any remuneration, and the procedures involved will be free of charge.

If any complications or damage happen due to your participation in the study, you will receive all necessary care free of charge.

**Patient Identifiers:
 (TAG)**

Data collected during the study will always be treated confidentially. The results will be presented jointly, without the identification of the participants, that is, your name will not appear in the publication of the results.

If you have any questions, you can contact the responsible researcher Eneida Rejane Rabelo da Silva, by phone (51) 9806 8616, the researchers Iasmin Borges Fraga (51) 98499 6318 and Larissa Gussatschenko Caballero (51) 9966 9496, also by phone, or the Research Ethics Committee of the Hospital de Clínicas de Porto Alegre (HCPA) by email (cep@hcpa.edu.br), phone (51) 3359 6246, or at the address Av. Protásio Alves, 211 – Portão 4, Bloco C, 5th floor – Rio Branco, Porto Alegre, RS, from Monday to Friday, from 8 a.m. to 5 p.m.

This Form is signed in two copies, one for the participant and one for the researchers.

**To be filled by the PARTICIPANT OR GUARDIAN:**

Name of the Participant/Guardian:______________________________________

Signature:_________________________________________________

*If patients are unable to write, insert their fingerprint stamp in the box on the side.

**To be filled by the IMPARTIAL WITNESS**:**

Name of the impartial witness:_____________________________________________

Signature:______________________________________________________________

**A witness is required if the participant is unable to read (for example, if blind or illiterate). The witness shall participate in any discussion about the participant’s consent. By signing this form, the witnesses guarantee that its information was explained to the participant, that they understood what was explained, and that they decided to participate in the study of their own free will.

**To be completed by the PROJECT TEAM:**

Name of the researcher who applied the form:_________________________________

Signature:______________________________________________________________

Porto Alegre, ___________________, 2023
